# Maternal vitamin A levels during second and third trimester and associations with offspring’s birth weight: a longitudinal cohort *post-hoc* study

**DOI:** 10.3389/fnut.2026.1835994

**Published:** 2026-06-08

**Authors:** Ilze Dirnena-Fusini, Signe Nilssen Stafne, Miriam Katarina Gustafsson, Mats Peder Mosti, Md Abu Jafar Sujan, Unni Syversen, Astrid Kamilla Stunes

**Affiliations:** 1 Department of Clinical and Molecular Medicine, Faculty of Medicine and Health Sciences, Norwegian University of Science and Technology, Trondheim, Norway; 2 Clinic of Rehabilitation, St. Olavs Hospital, Trondheim University Hospital, Trondheim, Norway; 3 Department of Public Health and Nursing, Faculty of Medicine and Health Sciences, Norwegian University of Science and Technology, Trondheim, Norway; 4 Regional Advice Office ALIS and SamLIS Midt, Trondheim Municipality, Trondheim, Norway; 5 Department of Research and Development, Clinic of Substance Use and Addiction Medicine, St. Olav’s Hospital, Trondheim University Hospital, Trondheim, Norway; 6 Department of Endocrinology, St. Olavs Hospital, Trondheim University Hospital, Trondheim, Norway; 7 Center for Oral Health Services and Research, Mid-Norway (TkMidt), Trondheim, Norway

**Keywords:** birth weight, maternal serum retinol, pregnancy, vitamin A deficiency, vitamin A insufficiency

## Abstract

**Background:**

Vitamin A is vital for maternal and fetal health during pregnancy, with both excess and deficiency linked to adverse outcomes. This study examined maternal vitamin A by serum retinol levels in the second and third trimesters and their association with birthweight in a high-income setting.

**Methods:**

This study is a *post-hoc* analysis of a merged dataset from 723 women with low-risk pregnancies in Norway. Clinical data and blood samples were collected in the second (week 18–22) and third trimester (week 32–36). Serum retinol was measured by high-performance liquid chromatography and offspring’s birthweight was collected from medical records. Regression analyses were performed to examine associations between retinol levels and offspring’s birth weight.

**Results:**

None of the women had vitamin A deficiency (serum retinol ≤ 0.7 μmol/L) in the second trimester compared to 1.9% in the third trimester. Vitamin A insufficiency (retinol > 0.7–≤ 1.0 μmol/L) was present in 8.6 and 43% of the women in the second and third trimesters, respectively. Overall, 17.3% of the offspring had a birthweight ≥ 4,000 g and were classified as having macrosomia. Mothers of macrosomic offsprings had lower serum retinol levels in the third trimester and lower average levels across both trimesters compared to mothers of offspring adequate for gestational age. A 0.2 μmol/L decrease in maternal serum retinol, measured in the second, third, as an average of both trimesters and as the delta change between trimesters, was associated with an increase in birth weight ranging from 31 to 68 grams.

**Conclusion:**

Vitamin A insufficiency was prevalent during the third trimester of pregnancy in a high-income setting. Maternal retinol levels were inversely associated with the offsprings’ birth weight and suggest that low maternal vitamin A levels may be associated with an increased risk of obesity in offspring. Considering the limitation of this study’s *post-hoc* design, these findings should be interpreted cautiously.

**Trial registration:**

http://clinicaltrials.gov, Identifier, NCT00476567.

## Introduction

According to the World Health Organization (WHO), vitamin A deficiency (VAD) remains a major public health concern globally, affecting more than half of all countries, with the highest prevalence occurring in low-income regions of Africa and South-East Asia (1, 2). The most severe effects of VAD are seen in pregnant women and young children, and according to the WHO Global Database on Vitamin A Deficiency (1995–2005), an estimated 15–30% of pregnant women in low- and middle-income countries had serum retinol levels corresponding to VAD (3). The most recent global estimate in children based on serum retinol (2013), showed that 29% of children aged 6–59 months worldwide were VAD (4). In high-income countries, vitamin A intake is generally considered adequate, and most research on VAD has focused on low-income regions. However, a 2019 study from US, reported that more than 60% of low-income, pregnant women with obesity had insufficient serum retinol levels in the third trimester (5).

During pregnancy, vitamin A is essential for maternal health and for the fetus’s normal growth and development, and maternal VAD increases the risk of adverse health outcomes (6, 7). Vitamin A is essential for the development of the visual system, and VAD remains the leading cause of preventable blindness in children (3). VAD in children increases the risk of death from infections in early life, and if VAD continues throughout childhood, it weakens the immune system, lowers survival, and increases long-term vulnerability to infectious diseases, limiting healthy development into later childhood (4). Maternal VAD has additionally been associated with anemia in both mother and offspring at delivery (8, 9). The demand for vitamin A is especially high in the third trimester due to the acceleration of fetal development (10), and both deficiency and excess can cause skeletal defects in the fetus (11).

Growing evidence indicates that vitamin A and its metabolites participate in key metabolic processes, including glucose and lipid metabolism, adipocyte differentiation and programming, and the regulation of body weight (12–15). These mechanisms may also play a role in fetal development and growth. Although adequate intake of vitamin A during pregnancy is mandatory, few studies have addressed the association of maternal vitamin A levels during pregnancy and offspring’s birth weight, especially in high-income settings. One study showed that elevated maternal vitamin A levels in mid-pregnancy were associated with an increased risk of low birth weight (16), while another found that high maternal vitamin A concentrations throughout pregnancy were positively associated with low birth weight and inversely associated with macrosomia in the second and third trimesters (17). A Mendelian randomization study including more than 200,000 individuals of European ancestry further demonstrated a strong association between maternal retinol levels and birth weight (18).

Birth weight is independently associated with long-term health outcomes. Both those born large for gestational age or macrosomic as well as those born with low birth weight (LBW) have an increased risk of cardiovascular disease, diabetes, and obesity later in life (19). Macrosomia in particular, is strongly associated with childhood obesity (20).

This study aimed to expand knowledge on vitamin A status during pregnancy in a high-income population. Specifically, it examined whether suboptimal, but not clinically deficient, vitamin A levels affected fetal growth and whether any such associations differed by trimester.

## Methods

### Study design and population

This *post-hoc* study is based on the merged data from all participants enrolled in the randomized controlled trial (RCT) “Training in Pregnancy” (TRIP) study, conducted in Norway between 2007 and 2009 (21). The aim in the original trial primarily was to evaluate the effect of regular exercise in pregnancy on the incidence of gestational diabetes. Women in gestational week 18–22 were randomly assigned to either a 12-week exercise program (intervention group) or standard antenatal care (control group), as previously described (21). Altogether 855 pregnant, Caucasian Norwegian women with low-risk pregnancy from two Norwegian cities, Trondheim (*n* = 660) and Stavanger (*n* = 195), were included. Inclusion criteria: pregnant women ≥ 18 years who attended the routine ultrasound at gestational week 18, with a singleton live fetus at the routine ultrasound scan and residing less than 30 min’ drive from the hospital. Exclusion criteria: high-risk pregnancies and/or conditions in which exercise training is contraindicated according to The American College of Obstetricians and Gynecologists (ACOG) Committee opinion (22).

### Data collection

The participants were recruited consecutively, and clinical data and overnight fasting blood samples were collected in the second and third trimesters (gestational weeks 18–22 and 32–36, respectively). Body weight (kg) and height (cm) were measured, and body mass index (BMI, kg/m^2^) was calculated. Blood pressure and glucose and glucose tolerance test were measured at both assessments in the second and third trimesters, as previously described (21). Questionnaires regarding pre-pregnancy body weight, sociodemographic variables, parity and lifestyle factors were completed, and pre-pregnancy BMI (p-BMI) was calculated (21). A validated, self-administered optical mark readable Food Frequency Questionnaire (FFQ) containing 180 food items (23), was used to collect information about diet and supplements in both trimesters. The FFQ was based on previous national dietary surveys, and intake was calculated by combining reported frequency and portion sizes, with household measures converted to grams using Norwegian standard portions (23, 24). The women were instructed to provide information about their dietary intake as it was the previous four weeks (24). Vitamin A intake was reported as Retinol Activity Equivalents (RAE), calculated as dietary retinol plus one twelfth of the estimated *β*-carotene intake (25, 26). The offspring’s birth weight and gestational age at delivery were collected from medical journals.

In this *post-hoc* study, participants allocated to the intervention or the control groups of the original RCT (21), were combined for the analyses. This was done as the two groups were similar in terms of the primary exposure (serum retinol), at both inclusion and post-intervention, and were also comparable with respect to the main outcome (birthweight). Data from participants from the two cities were also merged, as no differences in main exposure and main outcome were observed between the two study sites.

### Serum analyses

Fasting blood samples were collected at the inclusion in the second trimester and at assessment in the third trimesters (21, 27). Serum samples were stored at −80 °C until vitamin A (all-trans retinol) analyses were performed simultaneously in all samples by high performance liquid chromatography (HPLC), at the Department of Biochemistry, St. Olavs Hospital, Trondheim University Hospital, Norway.

### Classification of gestational hypertension and diabetes

Gestational hypertension was defined as systolic blood pressure > 140 mm Hg and/or diastolic blood pressure > 90 mm Hg in women with no pregestational hypertension, as previously described (21). The criteria for gestational diabetes were: fasting glucose in whole blood ≥ 6.1 mmol/L, or plasma glucose ≥ 7.0 mmol/L, or 2-h glucose level ≥ 7.8 mmol/L after oral glucose tolerance test in women with no pregestational diabetes, according to the guidelines that applied in 2007–2009 (21).

### Classification of serum vitamin A status

For vitamin A, we classified serum retinol concentrations according to established cut-offs: ≥ 2.45 μmol/L as excessive, ≥ 1.05–< 2.45 μmol/L as sufficient, ≥ 0.70–< 1.05 μmol/L as insufficient, and > 0.35–< 0.70 μmol/L as deficient (3). The same classification is applied for pregnant women (28), and was also used in the current study, with a small adjustment, as maternal serum retinol levels were reported with only one decimal from the biochemical analyses. Hence, for the distribution in groups, cut-offs were done for only first decimal and participants with serum retinol ≤ 1.0 μmol/L were included in the insufficient group.

### Classification of birth weight

Birth weight was classified in accordance with WHO recommendations and independently of gestational age (29–31). Infants were categorized as having low birth weight (LBW, < 2,500 grams), normal birth weight (NBW, ≥ 2,500–< 4,000 grams) or macrosomia (≥ 4,000 grams) (29–31).

### Classification of body mass index and gestational weight gain

Preconception BMI (p-BMI, kg/m^2^) was classified according to WHO as underweight (BMI ≤ 18.5 kg/m^2^), normal weight (> 18.5–< 25.0 kg/m^2^), overweight (≥ 25.0–< 30.0 kg/m^2^) and obesity class I and II (≥ 30.0–< 40 kg/m^2^) (32).

According to the Institute of Medicine (IOM) 2009 guidelines (Supplementary Table S1) (33), the recommended average weight gain during second and third trimester of pregnancy is 510 g/week if p-BMI is classified as underweight; 420 g/week if normal weight; 280 g/week if overweight and 220 g/week if obese class I and II (33).

### Statistics

Normality of data distribution and homogeneity of variance were tested by Shapiro–Wilk test and Levene’s test for equality of variance, respectively. Differences between the second and third trimesters were analyzed by a paired Student’s *t*-tests. Differences in maternal retinol between birth weight groups were analyzed by Kruskal-Wallis test with Dunn’s multiple comparison test. To determine the relationships between the intake of RAE and maternal serum retinol and BMI and retinol, Pearson correlation coefficients (r) were computed.

Linear regression models were employed to evaluate the associations between maternal serum retinol levels (measured during the second trimester, third trimester, as the average of both trimesters and as the change from the second to third trimester), and offspring birth weight. Confounders were chosen based on whether they were previously described to be associated with the outcome (34) or if they were statistically significant in the single linear regression analyses (Supplementary Table S2). The following regression models were performed: Crude model; Model 1, adjusted for maternal age, pre-pregnancy BMI, and gestational weight gain [second trimester (A) or third trimester (B, C, D)]; Model 2, Model 1 plus RAE intake [second trimester (A), third trimester (B), or mean (C, D)]; and Model 3, Model 2 plus randomized group allocation from the original study (control and intervention) and study site. Statistical analyses were performed using SPSS Statistics Version 26.0 (Armonk, NY: IBM Corp.), STATA/MP version 16.1 (StataCorp LP, College Station, TX, United States) and Microsoft Excel version 2,312 (Microsoft 365). Significance level was set at *p* ≤ 0.05.

## Results

### Participants

**Figure 1:**
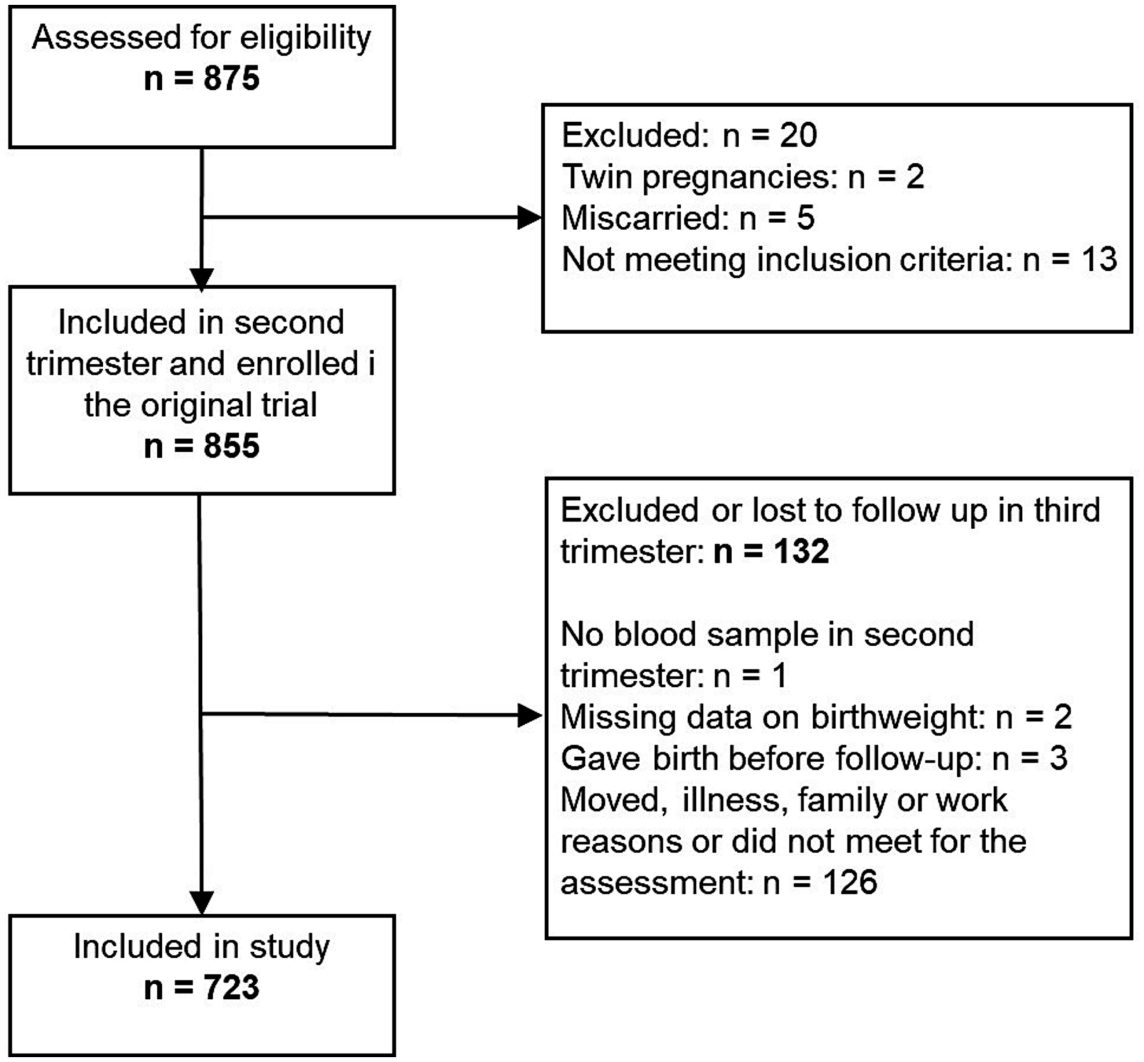
Flow chart of study.

A total of 875 women provided informed consent to participate in the original trial. Of these, 20 were excluded: 13 did not meet the inclusion criteria, five experienced miscarriages, and two had twin pregnancies, yielding a final sample of 855 eligible pregnant women included in the original study (21). For the present analysis, data on the primary exposure (second and third trimester serum retinol) and the primary outcome (birthweight) were not available for 132 participants. Therefore, the current analyses are based on data from 723 mother and offspring pairs. A flow chart of the study participants is shown in Figure 1. Baseline demographic and clinical characteristics of the population at inclusion in the second trimester (gestational week 18–22) are shown in Table 1.

**Table 1:** Characteristics of the study participants at inclusion in the second trimester.

Second trimester (gestational week 18–22), *n* = 723	Mean ± SD or n (%)
Age, years	30.5 ± 4.2
Gestation length, weeks	20.0 ± 1.7
Marital status^a^	
Married/Partner	706 (97.8)
Single	16 (2.2)
Education level and occupational status	
High School / Other	81 (10.8)
University	645 (89.2)
Paid work or self-employed^a^	681 (94.3)
Parity	
0	421 (58.2)
≥ 1	302 (41.8)
Smoking	6 (0.8)
p-BMI, kg/m^2,b^	25.1 ± 3.1
Inclusion BMI, kg/m^2,c^	24.7 ± 3.1
Gestational hypertension^#^	5 (0.7)
Gestational diabetes^##,d^	5 (0.7)
Vitamin A intake, RAE, μg/day	1,332 ± 656
Vitamin A intake, RAE < 770 μg/day	121 (16.7)

Data are in mean ± standard deviation (SD), or number (n) with percentage (%). ^a^Missing data from *n* = 1. ^b^Missing data from *n* = 3. ^c^Missing data from *n* = 2. ^d^Missing data from *n* = 16. p-BMI, pre-pregnancy body mass index; RAE, retinol activity equivalents. ^#^Gestational hypertension: systolic blood pressure > 140 mm Hg and/or diastolic blood pressure > 90 mm Hg. ^##^Gestational diabetes: fasting glucose in whole blood ≥ 6.1 mmol/L, or plasma glucose ≥ 7.0 mmol/L, or 2-h glucose level ≥ 7.8 mmol/L after an oral glucose tolerance test.

The mean gestational week at inclusion was 20.0 weeks, and the mean age was 30.5 years. Most women (89.2%) had higher education, and 58.2% had not given birth previously. Smoking during pregnancy was reported by 0.8% of the women. Mean p-BMI was 25.1 ± 3.1 kg/m^2^ and 1.7% were underweight, 77.9% had normal weight, 16.8% were overweight, 3.6% had obesity class I weight and had obesity class II weight before pregnancy (Supplementary Table S3).

The mean BMI at inclusion was 24.7 ± 3.1 kg/m^2^, and few of the women had gestational hypertension (0.7%) or gestational diabetes (0.7%).

The mean daily vitamin A (RAE) intake in the second and third trimesters was 1,322 ± 659 μg and 1,245 ± 589 μg, respectively. Vitamin A intake lower than the recommended 770 μg/day (35), was reported by 16.7 and 20.1% of the women in the second and third trimester, respectively.

### Maternal serum retinol levels

Mean serum retinol level during the second trimester was 1.34 ± 0.24 μmol/L (range: 0.70–2.40 μmoL/L), significantly declining to 1.09 ± 0.25 μmol/L (range: 0.50–2.20 μmol/L) in the third trimester (Table 2). None of the participants had serum retinol concentrations exceeding the upper reference limit of 3.0 μmol/L (3) in either trimester. VAD was not observed in the second trimester, whereas 9.3% of the women had vitamin A insufficiency. By the third trimester, 1.9% of the women had developed VAD, and 43.0% had vitamin A insufficiency (Table 2).

**Table 2:** Serum retinol levels in the second and the third trimester of pregnancy.

Serum retinol, μmol/L	n (%)	Mean ± SD
Second trimester		
Total	723 (100)	1.35 ± 0.24
Deficiency, serum retinol < 0.7 μmol/L	-	-
Insufficiency, serum retinol ≥ 0.7 – ≤ 1.0 μmol/L	62 (8.6)	0.95 ± 0.08
Sufficiency, serum retinol > 1.0 – ≤ 3.0 μmol/L	661 (91.4)	1.39 ± 0.21
Third trimester		
Total	723 (100)	1.09 ± 0.25*
Deficiency, serum retinol < 0.7 μmol/L	14 (1.9)	0.56 ± 0.05
Insufficiency, serum retinol ≥ 0.7 – ≤ 1.0 μmol/L	311 (43.0)	0.89 ± 0.11
Sufficiency, serum retinol > 1.0 – ≤ 3.0 μmol/L	398 (55.1)	1.26 ± 0.18

Data in mean ± standard deviation (SD) or number (n) with percentage (%). *p < 0.001 vs. mean retinol in the second trimester.

Among the women who had sufficient vitamin A levels in the second trimester 40% experienced a decline to insufficient levels, and 0.5% declined to VAD by the third trimester. Of the women with insufficient vitamin A levels in the second trimester, 8% improved to sufficient levels, while 18% declined further to VAD in the third trimester.

Since we in this study used a cut-off of 1.0 instead of 1.05 μmol/L for vitamin A insufficiency, the prevalence may have been underestimated. When using 1.1 μmol/L as a cut-off, the prevalence of vitamin A insufficiency increased to 19.1% (*n* = 138) and 62.2% (*n* = 450), in the second and third trimesters, respectively.

There was no significant correlation between maternal RAE intake (μg/day) and serum retinol (μmol/L) in the second trimester (*r* = 0.01, *n* = 723, *p* = 0.729), however maternal RAE intake showed a weak positive correlation with serum retinol in the third trimester (*r* = 0.09, *n* = 722, *p* = 0.013). The retinol levels and intake in both trimesters from a subgroup of this population have previously been reported in a mother-and-child follow-up study (36).

### Offspring birth weight and maternal retinol

A total of 29 infants were born late preterm (gestational week 34–36), the remaining 694 infants were all born at term. In total, 2.4% of the offspring were classified as LBW, 80.3% as NBW, and 17.3% as macrosomic (Table 3). Mothers of macrosomic offspring had significantly lower serum retinol concentrations in the third trimester and in the average levels of second and third trimester, compared to mothers of LBW and NBW offspring (Table 3). Additionally, the reduction in retinol levels from the second to the third trimester was significantly greater in mothers of macrosomic offspring than in those of LBW offspring (Table 3).

**Table 3:** Serum retinol levels and third trimester weigh gain categories stratified by offspring birth weight groups.

*n* = 723	Total		LBW		NBW		Macrosomic	
	Mean ± SD	n (%)	Mean ± SD	n (%)	Mean ± SD	n (%)	Mean ± SD	n (%)
Birth weight, g	3,547 ± 481	723 (100)	2,256 ± 474	17 (2.4)	3,435 ± 332	581 (80.3)	4,242 ± 235	125 (17.3)
Serum retinol, μmol/L								
Second trimester	1.35 ± 0.24	723 (100)	1.37 ± 0.17	17 (2.4)	1.36 ± 0.24	581 (80.3)	1.33 ± 0.24	125 (17.3)
Third trimester	1.09 ± 0.25	723 (100)	1.21 ± 0.26	17 (2.4)	1.10 ± 0.24	581 (80.3)	1.03 ± 0.25*	125 (17.3)
Mean of second and third trimesters	1.22 ± 0.22	723 (100)	1.29 ± 0.18	17 (2.4)	1.23 ± 0.22	581 (80.3)	1.18 ± 0.23**	125 (17.3)
Changes from second to third trimester (delta)	−0.26 ± 0.20	723 (100)	−0.16 ± 0.26	17 (2.4)	−0.26 ± 0.20	581 (80.3)	−0.30 ± 0.19***	125 (17.3)
Weight gain categories^#^, *n* = 716								
Low	1.07 ± 0.24	305 (100)	1.28 ± 0.29	9 (3.0)	1.06 ± 0.23	250 (81.9)	1.06 ± 0.26	46 (15.1)
Recommended	1.10 ± 0.25	289 (100)	1.16 ± 0.23	5 (1.7)	1.12 ± 0.26	240 (83.1)	1.02 ± 0.24	44 (15.2)
High	1.09 ± 0.24	122 (100)	1.10 ± 0.17	3 (2.5)	1.12 ± 0.24	84 (68.8)	1.00 ± 0.24	35 (28.7)

Data are in mean ± standard deviation (SD) or number (n) with percentage (%). LBW, low birth weight (< 2,500 g); NBW, normal birth weight (≥ 2,500–< 4,000 g), and macrosomic (≥ 4,000 g). *p = 0.020 vs. LBW and *p* = 0.006 vs. NBW. **p = 0.046 vs. LBW and *p* = 0.025 vs. NBW. ***p = 0.028 vs. LBW. ^**#**^Weight gain during third trimester in accordance with Institute of Medicine (IOM) 2009 guidelines based on pre-pregnancy body mass index (33) (Supplementary Tables S1, S3, S4).

### Weight gain during pregnancy

The optimal weight gain in different p-BMI subgroups was calculated individually, based on the IOM guidelines (33). During the second trimester, 17.1% of the participants had the recommended weight gain, 75.5% had lower weight gain than recommended, and 7.4% of the participants had higher weight gain than the recommended (Supplementary Table S3). During the third trimester, 40.4% of the participants had the recommended weight gain, and 42.6 and 17.0% had lower and higher weight than the recommended, respectively (Supplementary Table S4).

To avoid unreliable results due to small subgroup sizes, we did not conduct statistical analyses of serum retinol level differences across third trimester weight gain categories stratified by offspring’s birth weight categories.

### Associations between maternal serum retinol and offspring birth weight

Linear regression analyses showed a significant inverse association between maternal serum retinol concentrations and offspring birth weight (Table 4 and Supplementary Figure S1). This association was observed when retinol was examined as an independent variable measured in the second trimester (A), the third trimester (B), as the average of the second and third trimesters (C), and the as the delta change from the second to the third trimester (D).

**Table 4:** Mean differences in offspring birth weight in grams per 0.2 μmol/L decrease in maternal serum retinol measured in the second trimester, the third trimester, the average of the second and third trimesters and as the change (delta) from the second to the third trimester.

Retinol decrease per 0.2 μmol/L	Mean diff. and 95% CI	*p*	n	Mean diff. and 95% CI	*p*	n
	Crude Model			Model 1		
A: Second trimester	31.6 [2.0–61.1]	0.037	723	44.0 [13.9–74.2]	0.004	718
B: Third trimester	68.9 [40.7–97.1]	< 0.001	723	75.2 [47.5–103.0]	< 0.001	717
C: Mean of second and third trimester	61.9 [30.1–93.8]	< 0.001	723	74.4 [43.0–105.7]	< 0.001	717
D: Delta changes from second to third trimester	57.0 [22.7–91.2]	0.001	723	49.0 [14.8–83.1]	0.005	717
	Model 2			Model 3		
A: Second trimester	44.6 [14.5–74.7]	0.004	718	42.5 [11.8–73.2]	0.007	718
B: Third trimester	78.3 [50.5–106.2]	< 0.001	716	77.5 [49.3–105.8]	< 0.001	716
C: Mean of second and third trimester	77.5 [46.0–108.9]	< 0.001	716	76.3 [44.2–108.4]	< 0.001	716
D: Delta change from second to third trimester	49.4 [15.3–83.6]	0.005	716	50.5 [16.3–84.7]	0.004	716

Mean diff., mean difference (unstandardized linear regression coefficient β); 95% CI, 95% confidence interval. Model 1: adjusted for maternal age, pre-pregnancy body mass index (kg/m^2^), and weight gain since pre-pregnancy. Model 2: Adjusted for Model 1 + retinol activity equivalents (RAE) in μg/day in second (A), third (B) and mean of second and third trimester (C and D), respectively. Model 3: adjusted for Model 1 + Model 2 + original RCT group allocation (control and intervention) and study site (city).

In crude models, a 0.2 μmol/L decrease in retinol was associated with a higher mean birth weight of 31.6 g (*β* = 31.6, 95% CI: 1.9–6.1, *F*(1,721) = 4.39, *p* = 0.037, R^2^ = 0.006), 68.9 g (*β* = 68.9, 95% CI: 40.7–97.1, F(1,721) = 22.9, *p* < 0.001, R^2^ = 0.031), 61.9 g (*β* = 61.9, 95% CI: 30.1–93.8, F(1,721) = 14.6, *p* < 0.001, R^2^ = 0.020), and 57.0 g (*β* = 57.0, 95% CI: 22.7–91.2, F(1,721) = 10.7, *p* = 0.001, R^2^ = 0.015), for A, B, C and D, respectively (*n* = 723).

After adjustment for maternal age, pre-pregnancy BMI, and respective gestational weight gain (Table 4, Model 1), the associations remained statistically significant and were of similar or greater magnitude: A (*β* = 44.0, 95% CI: 13.9–74.2, *F*(4,713) = 9.3, *p* = 0.004, adjusted R^2^ = 0.046, *n* = 718), B (*β* = 75.2, 95% CI: 47.5–103.0, *F*(4,712) = 15.8, *p* < 0.001, adjusted R^2^ = 0.076, *n* = 717), C (*β* = 74.4, 95% CI: 43.0–105.7, F(4,712) = 14.1, *p* < 0.001, adjusted R^2^ = 0.068, *n* = 717), and D (*β* = 49.0, 95% CI: 14.8–83.1, F(4,712) = 10.5, *p* = 0.005, adjusted R^2^ = 0.050, *n* = 717).

Further adjustment for dietary vitamin A intake (RAE, μg/day, Table 4, Model 2) did not alter the strength or direction of the associations: A (*β* = 44.6, 95% CI: 14.5–74.7, *F*(5,711) = 8.1, *p* = 0.004, adjusted R^2^ = 0.049, *n* = 718), B (*β* = 78.3, 95% CI: 50.5–106.2, *F*(5,710) = 13.4, *p* < 0.001, adjusted R^2^ = 0.080, *n* = 716), C (*β* = 77.5, 95% CI: 46.8–108.9, F(5,710) = 12.3, *p* < 0.001, adjusted R^2^ = 0.073, *n* = 716), and D (*β* = 49.4, 95% CI: 15.3–83.6, F(5,710) = 9.1, *p* = 0.005, adjusted R^2^ = 0.053, *n* = 716).

Finally, the associations remained robust in the fully adjusted model (Table 4, Model 3), which additionally accounted for the original RCT group allocation and study site: A (*β* = 42.5, 95% CI: 11.8–73.2, *F*(7,710) = 5.9, *p* = 0.007, adjusted R^2^ = 0.047, *n* = 718), B (*β* = 77.5, 95% CI: 49.3–105.8, *F*(7,708) = 9.6, *p* < 0.001, adjusted R^2^ = 0.078, *n* = 716), C (*β* = 76.3, 95% CI: 44.2–108.4, *p* < 0.001, R^2^ = 0.080, *n* = 716), and D (*β* = 50.5, 95% CI: 16.3–84.7, F(7,708) = 6.8, *p* = 0.004, adjusted R^2^ = 0.054, *n* = 716).

## Discussion

This *post-hoc* longitudinal study examined the vitamin A status by serum retinol during pregnancy among mostly well-educated, pregnant women with low-risk pregnancies in a high-income setting. We found that none of the women had vitamin A levels exceeding the upper reference limit, while 8.6% of the women had vitamin A insufficiency in the second trimester, increasing to 43.0% in the third trimester. Vitamin A deficiency was not observed in the second trimester, and only 1.9% of the women displayed vitamin A deficiency in the third trimester. Serum retinol level was lower in the third trimester among mothers with offspring large for gestational age compared to mothers with offspring born adequate or small for gestational age. A decrease in maternal serum retinol level, measured during the second trimester, third trimester, as an average of both trimesters, and as a change from second to third trimester, was associated with an increase in birth weight. The association was strongest for retinol levels in late pregnancy, or as average and delta change from second to third trimester.

Studies addressing vitamin A status in pregnant women are warranted (10), also from high-income countries. We found a high prevalence (43%) of vitamin A insufficiency in the third trimester, despite the cohort’s high education and income level. This may even be an underestimate, as we used a serum retinol cut-off of 1.0 μmol/L instead of the recommended 1.05 μmol/L.

Pregnancy VAD appears more prevalent in low-income settings than in our study. A Turkish study including 427 women, found that 50% had insufficient vitamin A levels in the third trimester, with ~23% having VAD (37), and a 2019 review reported VAD prevalence in pregnancy ranging from 10.6 to 34.8% across several low- and middle-income countries (10). In contrast, a study conducted in India, described that only 23% of women exhibited insufficient vitamin A levels, of which 3.5% had VAD during the third trimester (38).

The decline in serum retinol levels observed in our study is consistent with previous research, first reported in 1943 (39), and later confirmed by numerous studies. For example, a study from China involving 1,209 pregnant women found that serum retinol concentrations were lower in the third trimester compared to the first and second trimesters, with a corresponding increase in the prevalence of VAD across trimesters (40).

The decline in circulating retinol during pregnancy may result from physiological changes during pregnancy, such as hemodilution. Conversely, increased retinol-binding protein levels could elevate circulating retinol levels. These opposing effects may alter the relationship between liver stores and serum retinol, potentially leading to an overestimation of vitamin A insufficiency and deficiency in pregnancy (41, 42). The decrease of vitamin A in the third trimester might also be caused by the increased demand for vitamin A, due to the accelerated fetal development in this phase (43, 44). Interestingly, higher vitamin A levels are reported in umbilical cord blood than in the pregnant women with VAD (44). Increased intrauterine transfer of retinol is reported with maternal vitamin A deficiency and insufficiency compared to sufficiency, indicating a compensatory mechanism stimulated by maternal vitamin A inadequacy (45). To avoid VAD, supplementation is necessary to increase vitamin A levels in the mother’s blood, milk, and umbilical cord (43).

The findings of the present study contrasts with those of our previous study, which included 41 pregnant women from 1986–88, and reported a substantially higher mean retinol during pregnancy (1.66 μmol/L vs. 1.22 μmol/L in the current study) (46). This discrepancy might be attributed to alteration in the dietary intake of vitamin A between study periods. Notably, in 2001, the vitamin A content in cod liver oil, a commonly used supplement in Norway, was reduced by 75%, from 250 μg/mL 50 μg/mL retinol. Additionally, the mean daily fish consumption in Norway decreased from 41 g in 1986–88 to 37 g in 2007–09 (47).

Maternal BMI prior to pregnancy and weight gain during pregnancy were associated with increased serum retinol levels, consistent with findings in a previous study (48). This association may reflect the role of adipose tissue as a storage site for retinoids, which are mobilized and converted to the active metabolite retinoic acid on demand (49, 50).

In our study, 17.3% of the offspring were classified as macrosomic Consistent with previous findings (17), we observed that mothers of macrosomic offspring had lower serum retinol levels in late pregnancy than mothers with NBW offspring. Furthermore, mean retinol concentrations across the second and third trimesters were lower in mothers with macrosomic offspring. Supporting this, we identified inverse associations between birth weight and maternal retinol levels in the second and third trimesters, average retinol levels across both trimesters, as well as the change in retinol levels from the second to the third trimester. Maternal VAD at delivery has been reported to be associated with low birth weight (51). A report from 2024, using data from over 200,000 individuals with European ancestry, found that retinol levels were strongly associated with birth weight (18). In this study, the authors concluded that maintaining normal retinol levels during pregnancy may help prevent low birth weight (18). In the current study population, only 2.4% of the offspring were classified as LBW, which may explain why we did not observe lower maternal serum retinol levels in mothers of LBW offspring compared to those with NBW offspring. We propose that maintaining maternal serum retinol within the sufficiency range during pregnancy is important for reducing the risk of both low birth weight and macrosomia.

The observed inverse associations between offspring birth weight and maternal retinol levels measured in both the second and third trimesters, as well as with the mean retinol levels across trimesters and the change in retinol levels between them, remained or were strengthened when adjusting for maternal confounders. These associations were strongest for retinol levels in late pregnancy and for the mean across both trimesters and remained robust across all adjusted regression models. Results from our study contribute to the evidence for developmental origins of diseases hypothesis and may indicate that low maternal vitamin A can be one of the possible triggers for increased obesity development in offspring later life.

We have previously reported antenatal vitamin D status in the same population as the current study and found that a 41% of these well-educated women with low-risk pregnancies had vitamin D levels (serum 25(OH)D) < 50 nmol/L in the third trimester (27). There is a possibility that the combined vitamin A and vitamin D insufficiency in pregnancy can affect offspring’s birth weight and future health. Additionally, vitamin A insufficiency might disrupt metabolic and hormonal regulation, potentially promoting fetal adipogenesis and overgrowth (12, 13, 15).

### Strengths and limitations

The main strengths of this study include a large sample size, high follow-up rate, and blood sampling in both the second and third trimesters. Serum retinol was measured using HPLC, the gold standard method. Few studies have assessed vitamin A status in pregnant women in high-income countries, highlighting the relevance of our work. However, the *post-hoc* design of this study may limit its conclusions. Also, the study population was relatively homogenous, well-educated Caucasian women with high socioeconomic status and low-risk pregnancies, which may limit generalizability due to potential selection bias. Participation in the original trial may have been influenced by the exercise intervention, possibly attracting healthier individuals. Additionally, most participants did not exceed the recommended pregnancy weight gain, further limiting generalizability. While serum retinol is useful for detecting deficiency, it may not reflect marginal vitamin A status due to homeostatic regulation and limited correlation with intake or clinical symptoms (34, 52). Lastly, multiple comparisons increase the risk of false positives, so findings should be interpreted with caution.

## Conclusion

The *post-hoc* analyses study demonstrates that vitamin A insufficiency, defined as serum retinol levels between 0.7 and 1.0 μmol/L, is prevalent, affecting 43% of women in their third trimester of pregnancy, even in a high-income setting. We also found that mothers of larger offspring had lower retinol levels, and that maternal retinol levels were inversely associated with offspring’s birth weight. These inverse associations were observed for retinol levels in both the second and third trimesters, the average across both trimesters, and the change in retinol levels over time, also when adjusted for maternal confounder. The inverse associations were strongest for late pregnancy and mean retinol levels and remained consistent across all adjusted regression models. These findings derive from a *post-hoc* analysis and should therefore be interpreted with caution. Additional studies are required to validate and further substantiate the observed associations. However, these results suggest that maintaining adequate maternal serum retinol levels during pregnancy may help reduce the risk of high birth weight and potentially lower the offspring’s long-term risk for obesity.

## Data Availability

The original contributions presented in the study are included in the article/Supplementary material, further inquiries can be directed to the corresponding author.

## Supplementary material

SUPPLEMENTARY FIGURE S1 Mean differences in offspring birth weight in grams per 0.2 μmol/L decrease in maternal serum retinol measured in the **(A)**: second trimester, **(B)**: the third trimester, **(C)**: the average of the second and third trimesters and **(D)**: as the change (delta) from the second to third trimester. Mean differences are presented as unstandardized linear regression coefficients *β* with 95% confidence intervals. Model 1: adjusted for maternal age, pre-pregnancy body mass index (kg/m2), and weight gain since pre-pregnancy. Model 2: Adjusted for Model 1 + retinol activity equivalents (RAE) in μg/day in second, third and mean of second and third trimester, respectively. Model 3: adjusted for Model 1, Model 2 + original RCT group allocation (control and intervention) and study site (city).
