# Supplementary figures and images for: Maternal vitamin A levels during second and third trimester and associations with offspring’s birth weight: a longitudinal cohort post-hoc study
Source: Front Nutr. 2026 Jun 8;13:1835994. doi: 10.3389/fnut.2026.1835994 (PMC13285689; doi:10.3389/fnut.2026.1835994)

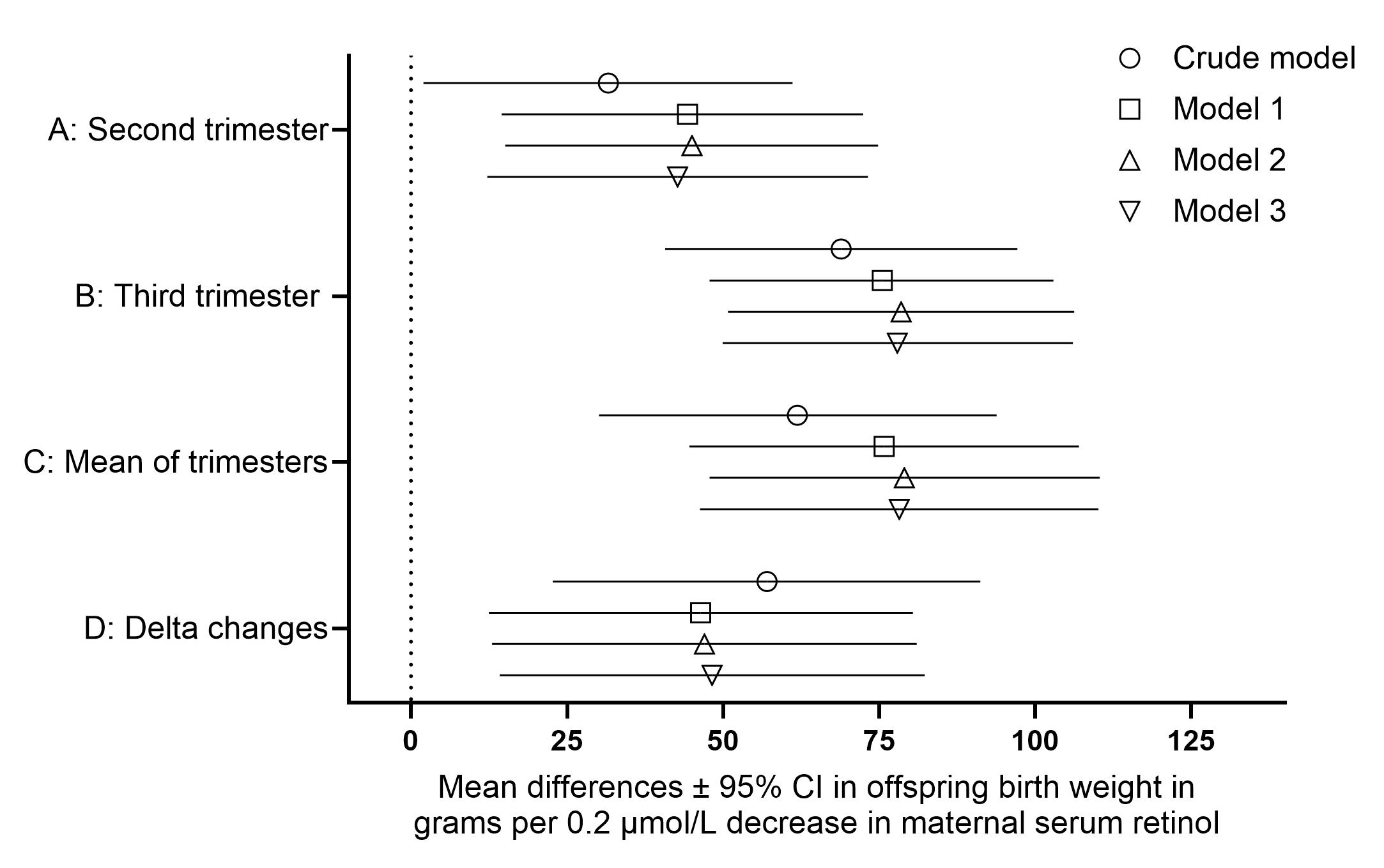

Supplement: SUPPLEMENTARY FIGURE S1 — Mean differences in offspring birth weight in grams per 0.2 μmol/L decrease in maternal serum retinol measured in the (A): second trimester, (B): the third trimester, (C): the average of the second and third trimesters and (D): as the change (delta) from the second to third trimester. Mean differences are presented as unstandardized linear regression coefficients β with 95% confidence intervals. Model 1: adjusted for maternal age, pre-pregnancy body mass index (kg/m2), and weight gain since pre-pregnancy. Model 2: Adjusted for Model 1 + retinol activity equivalents (RAE) in μg/day in second, third and mean of second and third trimester, respectively. Model 3: adjusted for Model 1, Model 2 + original RCT group allocation (control and intervention) and study site (city). [file Image_1.JPEG]
