# Supplementary material for: Maternal vitamin A levels during second and third trimester and associations with offspring’s birth weight: a longitudinal cohort post-hoc study
Source: Front Nutr. 2026 Jun 8;13:1835994. doi: 10.3389/fnut.2026.1835994 (PMC13285689; doi:10.3389/fnut.2026.1835994)
Supplement: Supplementary file 2 [file Table_1.DOCX]

**Supplementary Table S1. Gestational weight gain guidelines**

|  | **Total weight gain during pregnancy, kg** | **Incremental weight gain during the 2^nd^ and 3^rd^ trimester** |
| --- | --- | --- |
| **Pre-pregnancy BMI (p-BMI)** | **Range in kg** | **Range in kg per gestational week** |
| Underweight (< 18.5 kg/m^2^) | 12.5 –18.0 | 0.51 (0.44 – 0.58) |
| Normal weight (≥ 18.5 – < 25.0 kg/m^2^) | 11.5 – 16.0 | 0.42 (0.35 – 0.50) |
| Overweight (≥ 25.0 – < 30.0 kg/m^2^) | 7.0 – 11.5 | 0.28 (0.23 – 0.33) |
| Obese class I and II (≥ 30.0 kg/m^2^) | 5.0 – 9.0 | 0.22 (0.17 – 0.27) |

Recreated from Institute of Medicine 2009 Gestational Weight Gain Report [Institute of Medicine (US) and National Research Council (US). *Weight Gain During Pregnancy: Reexamining the Guidelines*. Rasmussen KM, Yaktine, AL, editors. Washington DC: The National Academies Press (2009).]
