# Supplementary material for: Maternal vitamin A levels during second and third trimester and associations with offspring’s birth weight: a longitudinal cohort post-hoc study
Source: Front Nutr. 2026 Jun 8;13:1835994. doi: 10.3389/fnut.2026.1835994 (PMC13285689; doi:10.3389/fnut.2026.1835994)
Supplement: Supplementary file 3 [file Table_2.DOCX]

**Supplementary Table S2. Univariate linear regression associations between mean change in offspring birth weight and maternal parameters**

|  | **β** | **Standard Error** | **p** | **95% Confidence Interval** | |
| --- | --- | --- | --- | --- | --- |
|  |  |  |  | **Lower** | **Upper** |
| **Vitamin A level, per μmol/L, 2^nd^ trimester, n = 723** | -157.81 | 75.33 | 0.037 | -305.70 | -9.61 |
| **Vitamin A level, per μmol/L, 3^rd^ trimester, n = 723** | -344.47 | 71.93 | < 0.001 | -485.68 | -203.25 |
| **Vitamin A level, per µmol/L, mean of 2^nd^ and 3^rd^ trimester, n = 723** | -309.56 | 81.15 | < 0.001 | -468.87 | -150.25 |
| **Vitamin A level, per µmol/L, decrease 2^nd^ to 3^rd^ trimester, n = 723** | -284.90 | 87.24 | 0.001 | -456.18 | -113.63 |
| **Age, years, n = 723** | 6.11 | 4.23 | 0.149 | -2.20 | 14.41 |
| **Pre-pregnancy BMI, n = 720** | 27.79 | 5.75 | < 0.001 | 16.50 | 39.07 |
| **Weight gain, pre-pregnancy to w. 18 – 22, kg, n = 718** | 18.53 | 6.90 | 0.007 | 4.99 | 32.08 |
| **Weight gain, pre-pregnancy**  **to w. 32 – 36, kg, n = 723** | 10.24 | 3.39 | 0.003 | 3.59 | 16.89 |
| **Vitamin A intake, RAE, µg/day, 2^nd^ trimester, n = 723** | 0.03 | 0.03 | 0.215 | -0.020 | 0.087 |
| **Vitamin A intake, RAE, µg/day, 3^rd^ trimester, n = 723** | 0.03 | 0.03 | 0.403 | -0.034 | 0.085 |
| **Vitamin A intake, RAE, µg/day, mean of 2^nd^ and 3^rd^ trimester, n = 723** | 0.05 | 0.03 | 0.190 | -0.022 | 0.112 |
| **Original study group allocation (exercise and ctr) n = 723** | 1.83 | 35.85 | 0.959 | -68.55 | 72.22 |
| **Study site (city), n = 723** | 53.12 | 45.0 | 0.238 | -35.23 | 141.46 |

Values represent unstandardized linear regression coefficient (β), Standard Error, p-value, and 95% Confidence Interval for mean change in offspring birth weight.
