# Supplementary material for: Maternal vitamin A levels during second and third trimester and associations with offspring’s birth weight: a longitudinal cohort post-hoc study
Source: Front Nutr. 2026 Jun 8;13:1835994. doi: 10.3389/fnut.2026.1835994 (PMC13285689; doi:10.3389/fnut.2026.1835994)
Supplement: Supplementary file 4 [file Table_3.DOCX]

**Supplementary Table S3. Weight gain during second trimester of pregnancy**

|  | **Gain, kg, mean ± SD** | **n** | **Low weight gain, n (%)** | **Recommended weight gain, n (%)** | **High weight gain, n (%)** |
| --- | --- | --- | --- | --- | --- |
| **Total** |  | 718 | 542 (75.5) | 123 (17.1) | 53 (7.4) |
| **p-BMI, kg/m^2^** |  |  |  |  |  |
| **≤ 18.5** | 3.8 ± 2.2 | 12 | 12 (100) | - | - |
| **> 18.5 – < 25.0** | 4.9 ± 2.8 | 559 | 451 (80.7) | 81 (14.5) | 27 (4.8) |
| **≥ 25.0 – < 30.0** | 4.2 + 3.0 | 121 | 65 (53.7) | 36 (29.8) | 20 (16.5) |
| **≥ 30.0** | 2.9 ± 3.3 | 26 | 14 (53.8) | 6 (23.1) | 6 (23.1) |

Weight gain categories at gestational week 20 based on pre-pregnancy body mass index (p-BMI) following the guidelines for Institute of Medicine recommended weight gain report [Institute of Medicine (US) and National Research Council (US). *Weight Gain During Pregnancy: Reexamining the Guidelines*. Rasmussen KM, Yaktine, AL, editors. Washington DC: The National Academies Press (2009)].
