# Supplementary material for: Maternal vitamin A levels during second and third trimester and associations with offspring’s birth weight: a longitudinal cohort post-hoc study
Source: Front Nutr. 2026 Jun 8;13:1835994. doi: 10.3389/fnut.2026.1835994 (PMC13285689; doi:10.3389/fnut.2026.1835994)
Supplement: Supplementary file 5 [file Table_4.DOCX]

**Supplementary Table S4. Weight gain during third trimester of pregnancy**

| **p-BMI,**  **kg/m^2^** | **Gain, kg, mean ± SD** | **n** | **Low weight gain, n (%)** | **Recommended weight gain, n (%)** | **High weight gain, n (%)** |
| --- | --- | --- | --- | --- | --- |
| **Total** |  | 716 | 305 (42.6) | 289 (40.4) | 122 (17.0) |
| **p-BMI, kg/m^2^** |  |  |  |  |  |
| **≤ 18.5** | 11.1 ± 3.3 | 12 | 10 (83.3) | 2 (16.7) | - |
| **> 18.5 – < 25.0** | 12.0 ± 3.7 | 557 | 267 (47.9) | 238 (42.7) | 52 (9.3) |
| **≥ 25.0 – < 30.0** | 11.3 ± 4.4 | 121 | 23 (19.0) | 40 (33.1) | 58 (47.9) |
| **≥ 30.0** | 9.0 ± 4.4 | 26 | 5 (19.2) | 9 (34.6) | 12 (46.2) |

Weight gain categories at gestational week 34 based on pre-pregnancy body mass index (p-BMI) following the guidelines for Institute of Medicine recommended weight gain report [Institute of Medicine (US) and National Research Council (US). *Weight Gain During Pregnancy: Reexamining the Guidelines*. Rasmussen KM, Yaktine, AL, editors. Washington DC: The National Academies Press (2009)].
